# 3D effects of a bone-anchored intra-oral protraction in treating class III growing patient: a pilot study

**DOI:** 10.1186/s40510-019-0290-0

**Published:** 2019-09-18

**Authors:** Mohammed Almuzian, Anas Almukhtar, Aman Ulhaq, Fahad Alharbi, M. Ali Darendeliler

**Affiliations:** 1 Faculty of Dentistry, University of Sydney, Sydney, Australia; 2 Edinburgh Dental Institute, University of Edinburgh, Edinburgh, UK; 3 College of Dentistry, University of Mosul, Mosul, Iraq; 4 University of Glasgow, Glasgow, UK; 5 Department of Preventive Dental Sciences, College of Dentistry, Prince Sattam Bin Abdulaziz, AL-Kharj, 11942 Saudi Arabia

**Keywords:** Alt-RAMEC, Miniscrews, Class III, Maxillary retrusion, Expansion, TADs

## Abstract

**Objectives:**

The aim of this prospective case series study is to assess the three-dimensional (3D) skeletal and soft tissue effects of the alternate rapid maxillary expansion and constriction (Alt-RAMEC) protocol in conjunction with a miniscrew-supported class III elastic wear in class III growing patients.

**Materials and methods:**

Fourteen consecutive participants (mean age 12.05 ± 1.09 years), who displayed class III malocclusions with retrognathic maxillae, were recruited. A cone beam computed tomography (CBCT) scan was acquired before commencing treatment (T1). All participants were treated with a tooth-bone-borne rapid maxillary expansion (hybrid MARME) appliance that was activated by the Alt-RAMEC protocol for 9 weeks. This was followed by full-time class III elastics, delivering 400 g/side, to protract the maxilla. When a positive overjet was achieved, protraction was ceased and a post-treatment CBCT scan (T2) was taken. The 3D analysis of pre- and post-treatment CBCT scans was blinded. The scans were registered on the anterior cranial base. The Euclidean distance between the two extracted surface models of the pre- and post-treatment scans was displayed as a color surface map.

**Results:**

All participants completed the intervention successfully. The majority of the participants showed protraction of the anterior maxillary region (71.4%) and in the zygomatic processes (64.2%). The maxilla significantly protracted (SNA 1.87° ± 1.06°; Vert.T-A 3.29 ± 1.54 mm), while the mandibular base significantly redirected posteriorly (SNB − 2.03° ± 0.85°, Vert.T-B − 3.43 ± 4.47 mm) and that was reflected on the ANB and Wits measurements. No adverse effects were observed.

**Conclusion:**

Class III elastics combined with the Alt-RAMEC activation protocol of the hybrid MARME appliance is an effective treatment method for mild/moderate class III malocclusions. A long-term follow-up and comparisons with other treatment modalities are required.

## Introduction

Treatment of class III malocclusion poses a challenge to the clinician. The timing of treatment varies from early intervention during the pre-pubertal stage of growth to late intervention after the cessation of facial growth. Part-time usage of protraction facemask (PFM) with maxillary expansion has been advocated as one of the efficient treatment modalities in the early treatment of class III malocclusion [1–5]. However, PFM therapy results in some dental effects including proclination of the maxillary incisors and retroclination of the mandibular incisors. The use of skeletal anchorage offers an encouraging alternative to optimize skeletal protraction with minimal dental side effects. Skeletal anchorage also eliminates the cumbersome need for an extra-oral appliance [6]. This could be achieved through the use of surgical plates [7] or mechanically retained temporary anchorage devices (TADs) [8, 9]; the former are placed under general anesthesia.

Rapid maxillary expansion (RME) in conjunction with PFM has been recommended to correct posterior crossbites and to disrupt the circummaxillary sutures [10], although the current evidence is limited with a high risk of bias [11, 12]. There are several designs for RME appliances including tooth-borne, tooth-tissue-borne, bone-borne, or hybrid types. However, it has been reported that bone-anchored RME can overcome the drawbacks associated with conventional tooth-borne and tooth-tissue-borne appliances, including tipping and periodontal damage of the anchor teeth [8, 9, 13]. Most recently, a new RME protocol was advocated for the treatment of class III malocclusions in cleft palate patients, in which the maxilla is alternately expanded and constricted in a weekly cycle over a period of 4–6 weeks [14–16]. The alternate rapid maxillary expansion and constriction (Alt-RAMEC) protocol has been demonstrated to produce a more pronounced “disarticulation” effect that allows for a significant amount of maxillary protraction in a considerably reduced amount of time [14–16].

While dento-skeletal, soft tissue, and airways outcomes can be analyzed using conventional two-dimensional (2D) cephalometric analysis [8, 17], the use of 3D methods provides a more representative assessment of treatment effects [17–19]. Positional changes can be assessed by measuring the Euclidean distance of the corresponding 3D points before and after treatment. Displaying post-treatment changes on the entire soft and hard tissue surfaces as a color-coded map is another acceptable way to present 3D analysis [20, 21].

The aim of this study is to assess the 3D skeletal and soft tissue effect of the Alt-RAMEC protocol in conjunction with TAD-supported class III elastic wear for protraction of the maxilla. The null hypothesis stated that the new protocol used for the treatment of class III malocclusion has no significant skeletal and soft tissue effects.

## Materials and methods

### Participants

The study was registered with the Australia New Zealand (ANZ) Clinical Trial Registry (ACTRN:12610000220066, ethical approval number: X10-010). The protocol was not published before the trial commencement. The data of this study were based on a previous study conducted by two of the authors (AD and MA) [8]. All participants from the treatment waiting list of the Orthodontic Department Faculty of Dentistry at the University of Sydney were screened. The inclusion criteria were (1) participants with a pre-pubertal stage of skeletal maturity and cervical vertebral maturational (CVM) stage II or III [22] and (2) participants with clinically diagnosed retrognathic or hypoplastic maxillae, anterior crossbites, and dental class III molars and canines without discrepancy between centric relation and centric occlusion (CR-CO). Participants with previous orthodontic/orthopedic treatment and with congenital abnormalities were excluded. Forty-two growing participants were identified with class III malocclusions. A senior clinician re-examined the participants to confirm the inclusion criteria. Only 14 patients (7 males and 7 females; 12.05 ± 1.09 years) out of the initial sample met the inclusion criteria. Pre-treatment radiographic images (T1) were taken in the natural head position by asking the patients to look into their own eyes in a mirror during the imaging procedure. Written informed consent was obtained from the parents or guardians.

### Treatment protocol [14, 23]

**Fig. 1:**
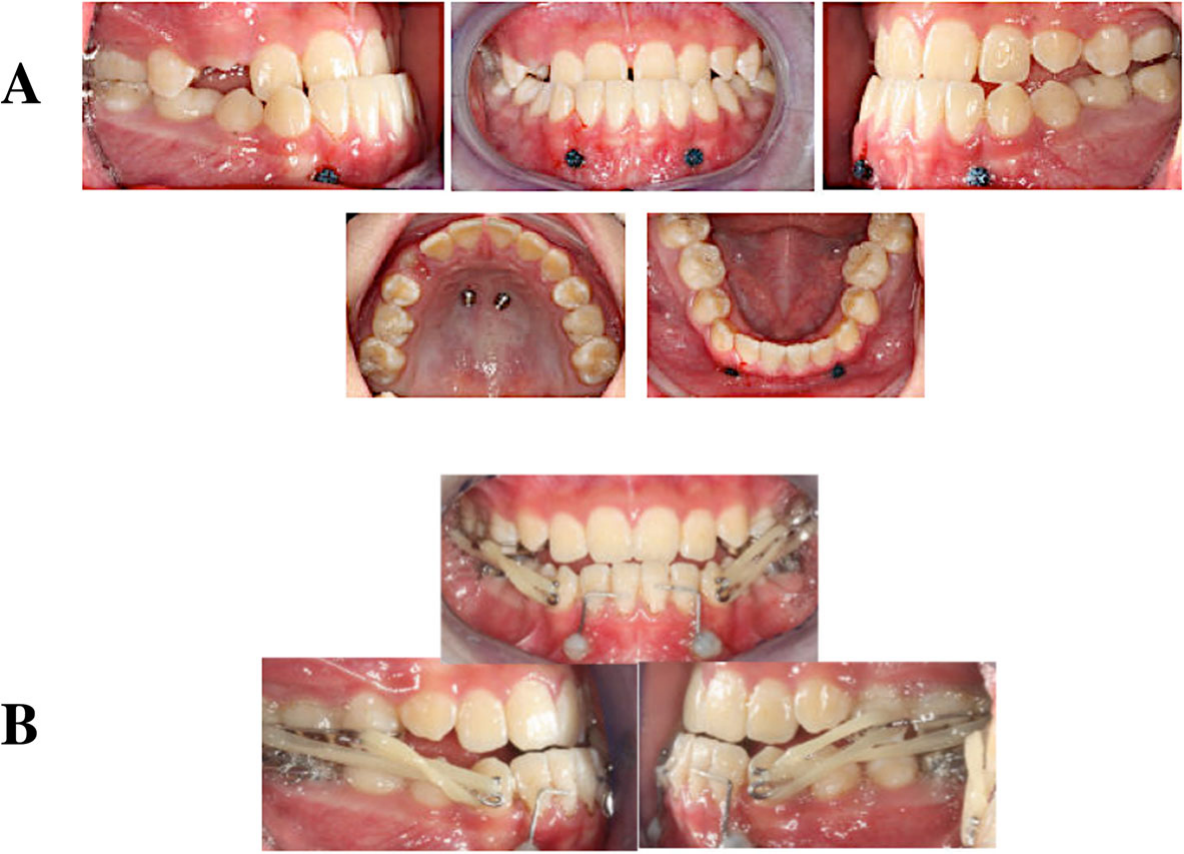
**a** Implant placement sites. **b** Appliances loaded with two elastics per side

**Fig. 2:**
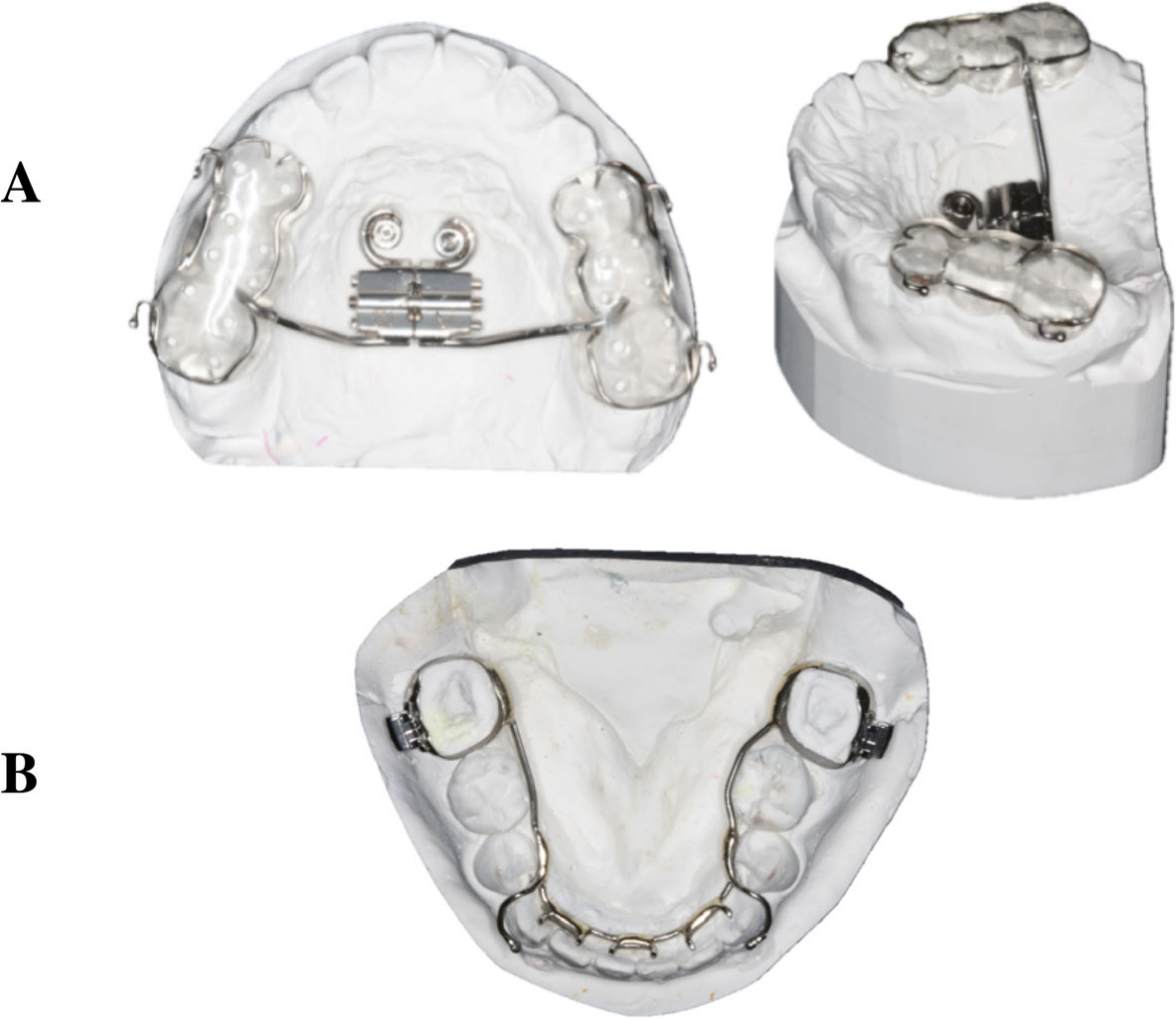
**a** Hybrid MARPE. **b** MLA appliance design

Two maxillary and two mandibular TADs were inserted following the insertion protocol (Table 1 and Fig. 1). One week later, molar bands were fitted around the lower first molars, and alginate impressions were then taken to construct a modified lingual arch (MLA). At the same visit, the palatal healing caps were removed and transfer impression copings were placed onto them for the subsequent transfer coping polyvinylsiloxane (PVS) maxillary impressions. After impression taking, the laboratory mini-implant analogs were positioned on the impression transfer abutments. The 3D relationships of the TADs in the oral cavity was thus duplicated on the plaster model. A hybrid micro-implant-assisted rapid maxillary expander (Hybrid MARME), using a macro-screw that produces 0.25 mm per quarter turn, was then constructed. Ball clasps (Remanium Ball Retainer Clasps, Dentaurum, Germany) were soldered to the appliance buccally at the region of the first premolars and first molars (Fig. 2). The Hybrid MARME was cemented with a glass ionomer cement (GIC) on day 28 of the TAD insertion. One mandibular TAD lost retention and was immediately replaced during the Alt-RAMEC phase.

**Table 1:** Skeletal anchorage systems

	Manufacturer	Size	Angulation	Insertion site	Specifications and requirement	Others
Mandibular TADs	Aarhus™ (MediconeG, American Orthodontics)	1.6 × 6 mm	30° apical angle	Between the mandibular canine and the lateral incisor labially	• Self-drilling • No irrigation	• Local anesthesia (2% lignocaine with 1: 80,000 adrenaline) was used • Pre-insertion swabbing with 0.12% chlorhexidine solution (Savacol, alcohol-free, Colgate). • Insertion was complete when the head of the TAD was flushed with the labial mucosa. • Postoperative daily use of 0.12% chlorhexidine solution (Savacol, alcohol-free, Colgate).
Maxillary TADs	Mondeal™ (GAC)	2 × 9 mm	90° angle	Anterior para-medial region	• Pre- (pilot) drilling of 1.5-mm holes • Surgical hand piece (speed 800 rpm) • Sodium chloride irrigation	

*rpm* round per minute

The MLA was constructed from 1-mm stainless steel wire (Remanium, Dentaurum, Germany) and cemented with GIC on day 28 after TAD insertion. The lingual cleats that extended from the MLA were bonded onto the lingual surfaces of the anterior teeth with a composite resin to hold the lower arch as one unit (Fig. 2). One participant had fractured buccal attachment of the MLA, and this was repaired during the protraction phase

All participants were instructed to expand the hybrid MARME by 1 mm/day for 7 days (two turns in the morning and two turns in the evening) [9]. One week later, all participants presented for expansion assessment; if satisfactory, the participants were then instructed to constrict the maxilla by unwinding the hybrid MARME by 1 mm/day for 7 days. This cycle was repeated until week 9. After 9 weeks of alternating expansion and contraction, the mobility of the maxilla was subjectively and manually assessed. This was done by supporting the forehead and bridge of the participant’s nose with one hand and holding the maxillary incisors with the other. The maxilla was then moved in an anterior and posterior direction to detect the mobility of the maxilla. When mobility “disarticulation” was detected, the second phase (the protraction phase) of treatment commenced.

On both sides, a 0.019 in. × 0.025 in. stainless steel (SS) wire was then bent to fit passively into the crossheads of the lower TADs and was secured with flowable composite to the labial surface of the lower incisors; the aim was to hold the lower dental unit to the bone through the lower TADs. Two full-time heavy intraoral elastics per side, producing a total of 400 g/side, were prescribed. The participant was instructed to replace the elastics once a day. Elastics ran in the long class III configuration, from the posterior ball clasps on the hybrid MARME to the “S” hook at the lower canine regions. This configuration was adopted to prevent the anticipated counterclockwise rotation of the maxilla.

The participants were then assessed at 2-week intervals until a + 2-mm overjet was achieved. Once the overjet was corrected, the appliances were removed, no retention appliances were provided, and post-treatment records were then taken (T2).

### 3D analysis

For each participant, a set of full-head (12 inches) pre-treatment and immediate post-treatment CBCT scans were captured using Newtom 3G (QR, Verona, Italy). These were carried out by experienced technicians following a standardized protocol at the Orthodontic Department at the Faculty of Dentistry at the University of Sydney. Both scans were captured with the appliance inserted and the mandible in the centric relation position. The voxel size was set at 0.4 mm, and the images were saved in DICOM format (Digital Imaging and Communications in Medicine).

The pre- and post-treatment DICOM images were blindly and simultaneously loaded to the OnDemand3D software (Cybermed Inc., Seoul, Korea). The post-treatment image for each patient was superimposed on the pre-treatment image of the same patient using the voxel-based registration method [24]. The target region for the superimposition was selected to include the forehead and anterior cranial base regions in which the algorithm searches for the best match between the grayscale intensity of the superimposed images voxel-by-voxel within the outlined region of interest; this was the anterior cranial base in our study.

The forehead and anterior cranial base regions were favored for superimposition because of its distance from the area of active treatment; thus, no change was expected in this region as a result of the treatment which allows it to be considered as a reliable reference to compare the treatment changes. The post-treatment image (superimposed) was then saved in its new position as a DICOM image file ready for the next step of the analysis.

The pre- and post-treatment images were loaded on the Maxilim software package (Medicim-Medical Image Computing, Belgium). For each image, the skeletal 3D model was extracted and saved as stereolithography (STL) file ready for assessment. The CBCT number (CN), equivalent to Hounsfield unit (HU) for CT scan, was standardized at 276 units for the segmentation of the skeletal models in all cases.

The superimposed pre- and post-treatment images were simultaneously loaded on VRMesh software package (VirtualGrid, Bellevue City, WA, USA). The Euclidian distances between the two images were displayed as a color-coded 3D image surface. Each vertex on the post-treatment image surface was given a specific color based on its distance from the nearest point on the superimposed pre-treatment image surface. The associated color scale was set to maximum (+ve) and (−ve) of 1 mm; this means that points that measure a positive distance equal and more than 1 mm were highlighted in a dark red color, and points that measures less than 1 mm were graded into different shades of lighter red, orange, then yellow colors ending with green color at zero distance. On the lower side of the scale, points that measures a negative distance equal and more than 1 mm were highlighted in a dark blue color, and points that measures less than 1 mm were graded into lighter shades of blue colors ending with green color at zero distance. The color-coded post-treatment image was saved for objective visual analysis. Areas selected for the analysis were the anterior surface of the maxilla and the zygomatic processes of the maxilla, anterior region of the mandible, and the inferior borders of the mandible.

## Results

The aim of the treatment intervention was achieved in all participants over a mean period of 8.5 weeks of protraction (range 8–9 weeks) with no significant adverse effects on the tooth roots, alveolar bones, and periodontal tissues.

**Fig. 3:**
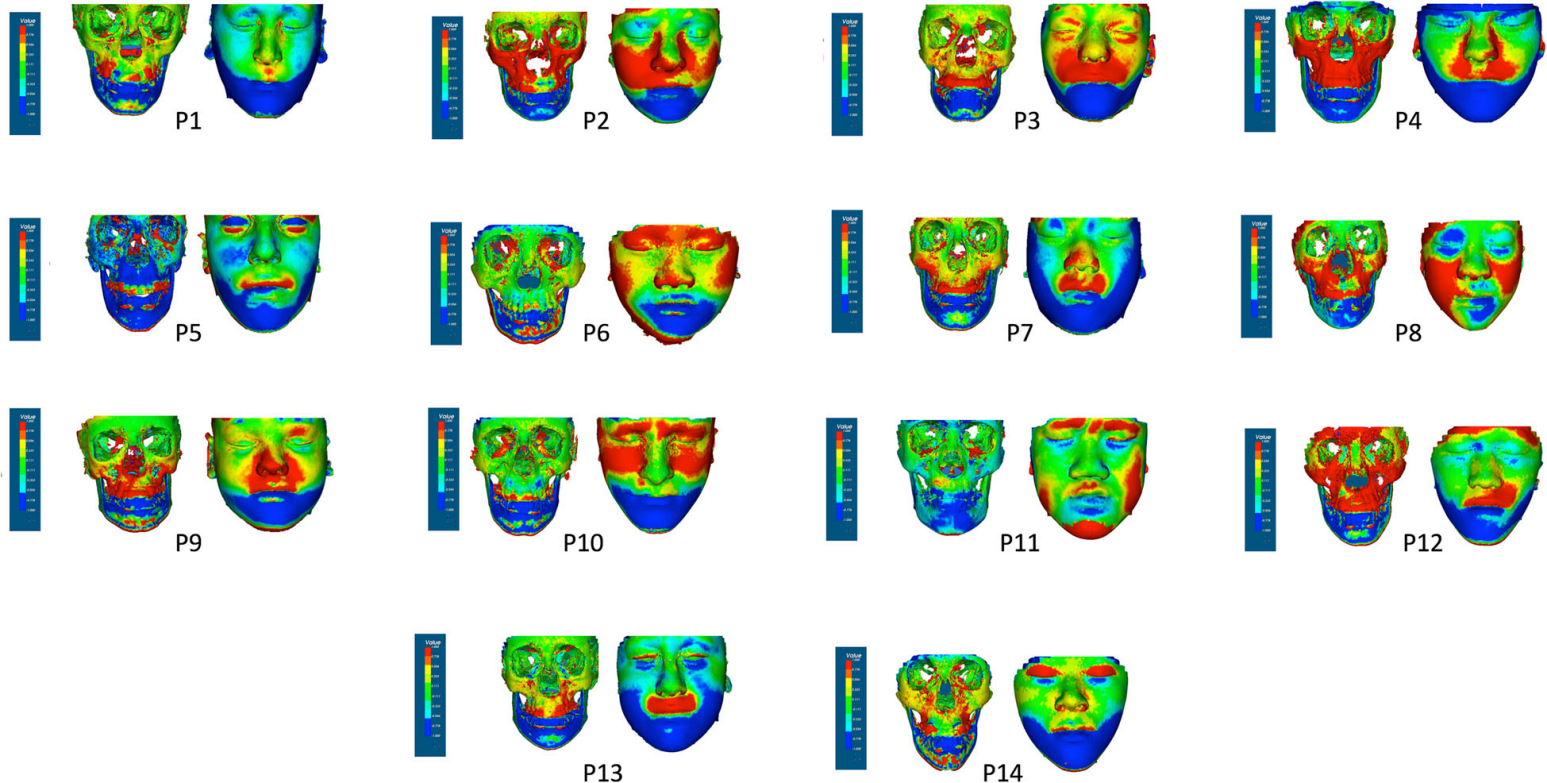
Color maps of the patients

Each patient was assessed for the changes after treatment in the following anatomical regions: anterior surface of the maxilla and zygomatic processes of the maxilla and anterior region of the mandible. The skeletal and soft tissue changes between T1 (pre-treatment) and T2 (immediate post-treatment) are shown as color maps in Fig. 3. The red color in the 3D mapping indicated an outward movement equal or more than 1 mm, blue color indicated inward movement of equal or more than 1 mm, and green color indicated no movement.

**Fig. 4:**
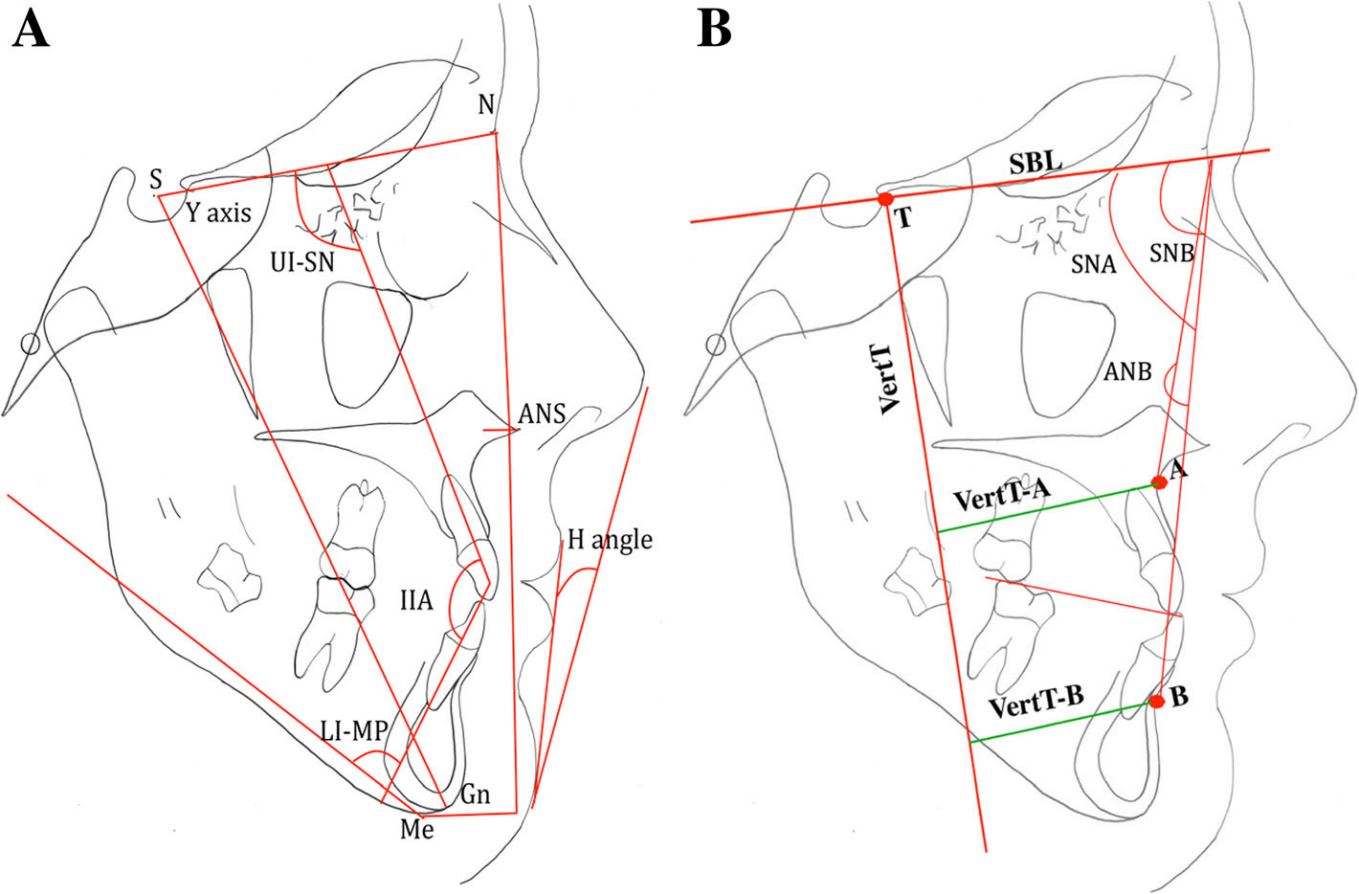
**a**, **b** Cephalometric variables used to evaluate the skeletal, dental, and soft tissue changes from T1 to T2 (SNA (°), Vert. T-A (mm), SNB (°) Vert. T-B (mm), ANB (°), WITTS appraisal (mm), mid-facial height (N-ANS) (mm), lower facial height (ANS-ME) (mm), upper facial height ratio (N-ANS/N-ME) (%), lower facial height ratio (N-ME/ANS-ME) (%), *y*-axis (°), upper incisors inclination (UI-SN) (°), lower incisors inclination (LI-MP) (°), inter-incisal angle (IIA) (°), overjet (OJ) (mm), overbite (OB) (mm), harmony (H), angle (n-me-ls) (°))

On the 3D analysis, most patients showed a positive (outward) movement in the anterior maxillary region (10 out of 14) and in the zygomatic processes. This was confirmed in the 2D cephalometric analysis in which the angular (Sella-Nasion to A (SNA) = 1.87 ± 1.06°) and linear (Vert.T-A 3.34 ± 1.54 mm) measurements of the anteroposterior position of the maxilla showed significant protraction (Table 2, Fig. 4).

**Table 2:** Skeletal, dental and soft tissue changes from T1 to T2

Variables	T1	T2		T2-T1				
	Mean	SD	Mean	SD	Mean	SD	*p* value	Significance
Anterioposterior changes								
SNA (°)	78.37	2.49	80.24	2.92	1.87	1.06	0.000	***
Vert. T-A (mm)	46.23	8.8	49.57	8.93	3.34	1.54	0.000	***
SNB (°)	82.11	3.19	80.09	3.53	− 2.02	0.85	0.000	***
Vert. T-B (mm)	39.57	14.69	36.14	12.95	− 3.43	4.47	0.013	*
ANB (°)	− 3.75	2.89	0.2	2.77	3.95	0.57	0.000	***
WITTS appraisal (mm)	-9.63	2.5	− 4.47	2.67	5.16	1.51	0.000	***
Vertical changes								
Mid-facial height (N-ANS) (mm)	52.27	2.99	54.95	2.35	2.68	1.53	0.447	NS
Lower facial height (ANS-ME) (mm)	69.44	4.76	72.63	5.34	3.19	2.21	0.000	***
Upper facial height ratio (N-ANS/N-ME) (%)	44.3	1.88	43.13	1.91	− 1.17	1.21	0.003	**
Lower facial height ratio (N-ME/ANS-ME) (%)	55.67	1.99	56.87	1.91	1.2	1.24	0.003	**
*y*-axis (°)	67.38	3.6	69.33	4.08	1.95	1.11	0.000	***
Dentoalveolar changes								
Upper incisors inclination (UI-SN) (°)	104.51	6.6	107.49	6.24	2.98	2.71	0.001	**
Lower incisors inclination (LI-MP) (°)	84.82	4.97	81.61	3.64	− 3.21	3.4	0.004	*
Inter-incisal angle (IIA) (°)	135.29	7.17	133.88	5.94	− 1.41	4.55	0.268	NS
Overjet (OJ) (mm)	− 2.89	1.41	2.74	1.11	5.63	1.36	0.000	***
Overbite (OB) (mm)	1.57	1.92	0.36	1.46	− 1.21	1.89	0.033	*
Soft tissue profile changes								
Harmony (H) angle (n-me-ls) (°)	6.36	4.47	9.12	3.97	2.76	1.8	0.0001	***

The changes in the anterior mandibular region showed a negative (inward) movement (12 out 14 patients). This was combined with downward displacement of the mandible shown as a red patch along the lower border of the mandible (14 out of 14). Again, these findings were confirmed in the 2D cephalometric analysis (Vert.T-B − 3.43 ± 4.47 mm, *p* < 0.05; Sella-Nasion to B (SNB) − 2.02° ± 0.85°, *p* < 0.001). Collectively, the maxillo-mandibular relationship improved as reflected in the ANB and Wits measurements, + 3.95° (± 0.57°) and 5.16 mm (± 1.5 mm), respectively (Table 2). At the dental level, changes involved significant proclination of the upper incisors (UI-PP = 2.98° ± 2.71°) and retroclination of the lower incisors (3.2° ± 3.4°) (Table 2). The combined dento-skeletal changes led to a significant improvement in the overjet (5.62 ± 1.36 mm) as revealed by cephalometric analysis (Table 2).

## Discussion

### Study findings

The aim of this study was to describe the 3D treatment changes of intra-oral protraction combined with Alt-RAMEC protocol in treating class III patients.

One of the growth patterns in class III cases is the dominant mandibular forward movement. The treatment intervention in this study intended to compensate for this pattern and correct the skeletal class III relationships by forward displacement of the anterior maxillary region. In most of the patients, as shown in 3D color mapping, there was a forward displacement of the anterior maxillary region and the zygomatic processes combined with negative (inward) movement or no changes at the anterior mandibular region and increase in the lower facial height as shown in 2D cephalometric analysis. These treatment effects come in agreement with previous studies that reported a favorable maxillary advancement in the Alt-RAMEC/FM group compared to the conventional RME/FM group [14, 23, 25] although both Isci et al. [25] and Fischer et al. (2018) used a different Alt-RAMEC protocol. In these studies, the patients were instructed to follow an Alt-RAMEC protocol that produced 0.4 mm of expansion (two turns of activation/day) for 4 and 6 weeks, respectively. In our study, the patients were instructed to follow a 9-week Alt-RAMEC protocol, turning the activation key four times/day producing 0.8–1 mm of expansion for 9 weeks. Despite the heterogeneity in the methodology between these studies, it seems that the amount of daily expansion (0.4 or 1 mm per day) and the duration of the Alt-RAMEC protocol (6 weeks or 9 weeks) induced insignificant differences; hence, the treatment changes of our protocol were in-line with those reported in the literature [26–28].

Similarly, the anterioposterior mandibular position was significantly improved secondary to the intervention, again probably due to the full-time utilization of the class III elastics and the disarticulation effect of the prolonged Alt-RAMEC protocol. Although one of the study inclusion criteria was to eliminate participants with a clinically detectable mandibular displacement, the authors acknowledge that there was a possibility of undetected shifts from the retruded centric position (RCP) to the intercuspal position (ICP). Hence, the argument might be made that the changes in the anterior mandibular region were surpassed as a result of the elimination of pre-treatment possible functional mandibular displacement secondary to the intervention, and this could be overcome by taking radiographical images in RCP. Nevertheless, it is important to acknowledge that taking a radiographical image at RCP is not immune from error for two reasons. First, as the ICP is a result of an engram (conditioned reflex of the neuromuscular system), this makes manual seating of the condyles into the RCP very difficult. Secondly, taking records in the RCP could induce another inherent pseudo-increase in the facial height.

Although the use of intermaxillary forces applied to the miniplates appears to be a promising treatment method to class III malocclusion as suggested in previous studies [9, 29], the placement of the miniplates requires an invasive surgical procedure. In our study, similar results were achieved using miniscrews without the need for more invasive surgery that requires general anesthesia. However, by comparing our results to Isci et al. [25] and Fischer et al. (2018), it seems that the addition of miniscrew to the Alt-RAMEC protocol did not provide superior results in terms of maxillary protraction.

A posterior rotation of the mandible and an increase in the anterior facial height are common biomechanical effects of the PFM treatment [27, 28, 30, 31]. Similar changes were observed in our study in the form of significant increases in the lower facial height. This was observed as a red line at the inferior border of the mandible indication downward repositioning of the mandible.

### Strengths and limitations of the study

One might argue that there were unusual changes at the forehead and orbital area of some patients. This might raise the following question: “Did these zygomatic and anterior maxillary regions move forward, or was the registration inaccurate?” To answer this question, we must first explain the voxel-based registration (VbR) superimposition method utilized in this study. VbR is the preferred method of registration for a number of reasons: (1) VbR relies on the grayscale intensity of the CBCT image voxels rather than the constructed 3D surface, this makes it more reliable than other methods when dealing with low-resolution and high-noise images; (2) because this study was designed to assess the effect on both the hard and soft tissues, it was logical and more reliable to use a method that registers both tissues simultaneously which could only be achieved using VbR [24]. Secondly, VbR deals with the DICOM image as one unit and performs the registration of the image relying on comparing the grayscale of the predominant tissue which in this study was the skeletal tissue leaving the discrepancies at the soft tissue boundaries to be ignored by the registration algorithm. In this study, it was crucial to have the skeletal tissue accurately registered; hence, the skeletal tissue at the areas around the eyes and the cranial base did not move and therefore represented by the green color. Now, having the skeletal tissue perfectly registered, the reason for the red color at the unexpected regions could be attributed to the changes over the time span between the two CBCT scans. In our study, the time was on average 8.5 weeks which is long enough for a teenage patient to gain or lose some weight at different regions of the face. For some patients, the soft tissue around the eyes and nose appears red while the skeletal tissue at the same region is still green. This indicates that the patient has grown or gained some weight (P5, P9, P11, P14) or there might have been a difference in the facial expression (P10) at the time of the scans. In addition, the color scale has been set up to 1 mm; this makes trivial changes in soft tissue as low as 1 mm to take a red or blue color and appear as positive or negative changes, respectively. It is not uncommon to see confusion between the soft and hard tissue changes especially with longitudinal 3D analysis studies of a considerable time scale.

Furthermore, the authors acknowledge the small sample size and lack of control group of this study to comment on the validity of the use of this novel approach in treating class III malocclusion compared to other established methods. Another limitation of this study is that the findings were evaluated using quantitative visual assessment of the color maps. Nevertheless, there are no available 3D norms to compare with the findings of this study. Another possible drawback is participant compliance with performing the expansion and constriction of the maxilla and the daily interchange of the elastics. Further studies are required on larger samples of treated and control subjects possibly with a randomized clinical trial design.

## Conclusion

Bone-anchored class III protraction, in conjunction with a MARME appliance and an Alt-RAMEC protocol, improves the maxillo-mandibular relationship in class III malocclusion. Short-term treatment effects include skeletal and soft tissue changes. A long-term randomized clinical trial with a larger sample size is recommended for verification.

## Data Availability

Data and materials are available at the Orthodontic Department in the Faculty of Dentistry, University of Sydney.

## Abbreviations

Alt-RAMEC: Alternate rapid maxillary expansion and constriction
ANZ: Australia New Zealand
CBCT: Cone beam computed tomography
CN: CBCT number
DICOM format: Digital Imaging and Communications in Medicine
Hybrid MARME: Hybrid micro-implant-assisted rapid maxillary expander
ICP: Intercuspation position
MLA: Modified lingual arch
PFM: Protraction facemask
PVS: Polyvinylsiloxane
RCP: Retruded centric position
RME: Rapid maxillary expansion
SS: Stainless steel
STL: Stereolithography
T1: Pre-treatment
T2: Immediate post-treatment
TADs: Temporary anchorage devices
VbR: Voxel-based registration
